# Characterizing the Moisture Content of Tea with Diffuse Reflectance Spectroscopy Using Wavelet Transform and Multivariate Analysis

**DOI:** 10.3390/s120709847

**Published:** 2012-07-23

**Authors:** Xiaoli Li, Chuanqi Xie, Yong He, Zhengjun Qiu, Yanchao Zhang

**Affiliations:** 1 College of Biosystems Engineering and Food Science, Zhejiang University, 866 Yuhangtang Road, Hangzhou 310058, China; E-Mails: xiaolili@zju.edu.cn (X.L.); xcq1130@yahoo.com.cn (C.X.); zjqiu@zju.edu.cn (Z.Q.); hylab@zju.edu.cn (Y.C.Z.); 2 Cyrus Tang Center for Sensor Materials and Applications, Zhejiang University, 866 Yuhangtang Road, Hangzhou 310058, China

**Keywords:** diffuse reflectance spectroscopy, moisture content, tea, wavelet transform, wavelength selection

## Abstract

Effects of the moisture content (MC) of tea on diffuse reflectance spectroscopy were investigated by integrated wavelet transform and multivariate analysis. A total of 738 representative samples, including fresh tea leaves, manufactured tea and partially processed tea were collected for spectral measurement in the 325–1,075 nm range with a field portable spectroradiometer. Then wavelet transform (WT) and multivariate analysis were adopted for quantitative determination of the relationship between MC and spectral data. Three feature extraction methods including WT, principal component analysis (PCA) and kernel principal component analysis (KPCA) were used to explore the internal structure of spectral data. Comparison of those three methods indicated that the variables generated by WT could efficiently discover structural information of spectral data. Calibration involving seeking the relationship between MC and spectral data was executed by using regression analysis, including partial least squares regression, multiple linear regression and least square support vector machine. Results showed that there was a significant correlation between MC and spectral data (*r* = 0.991, RMSEP = 0.034). Moreover, the effective wavelengths for MC measurement were detected at range of 888–1,007 nm by wavelet transform. The results indicated that the diffuse reflectance spectroscopy of tea is highly correlated with MC.

## 1. Introduction

Tea is produced from fresh burgeon of tea plant after a series of physical and chemical reactions in the various tea processing procedures. Generally speaking, the tea processing procedures are always accompanied with great variations of moisture content (MC). There are three main processing procedures including fixation, rolling and drying for green tea. The fixation procedure is implemented by high temperature processing to reduce the activity of enzymes, to eliminate herbaceous odor components, and to evaporate some water. Especially, the drying procedure dehydrates tea to reduce MC and to improve tea's smell and taste after thermochemical reactions under high temperature. Therefore, the MC of tea not only determines the shelf life of tea, but also affects the physical and chemical reactions in tea processing, so measurement of MC is an important task for producing high-quality tea [1].

The traditional way of accurately measuring MC is the gravimetric method, which takes several hours and cannot meet the requirements of real-time, on-line detection of MC in tea processing. Moreover, the gravimetric method reduces the quality of tea, so tea measured by this method usually has to be discarded.

Diffuse reflectance spectroscopy (DRS) measures the reflectance from the surface of study objects, but DRS does not involve exactly the surface, as most of the light is contributed by scattering centers beneath the surface. The reflectance attribute and its derivatives have been proven to be highly correlated with a number of physicochemical properties [2]. Recent improvement in visible/near infrared (Vis/NIR) spectroscopy have made DRS a convenient, simple, reliable and fast tool in quality evaluation and measurement of agricultural products and food. Vis/NIR can reflect the absorption characteristic of the main chemical bonds of C–H, N–H, O–H, so it has been widely used for quantitative analysis of compositions of organic substances [3]. Especially, the absorptivity of water (as O–H stretch) is relatively high compared with that of most other substances in Vis/NIR spectroscopy [4], so Vis/NIR diffuse reflectance spectroscopy may be a potential way for measurement of MC. Researchers have used the NIR technique to determinate MC of semolina pasta [5], foliage [6–8], black tea [9], green tea [10], soil [2], tuna fish [11] and crop [12], *etc.*, but the current research on tea only focuses on fresh leaves of tea plants or processed tea. Tea is produced from leaves through a set of physical and chemical reactions, which result in huge variations of MC, external morphology and internal composition of leaf, and these variations occur throughout the manufacturing process. Furthermore, the external and internal attributes of partially processed tea under heating and drying are greatly different from those of foliage under natural water stress, which may result in different spectral responses, so analysis of the relationship between MC and Vis/NIR diffuse reflectance spectroscopy of tea based only on fresh tea leaves or processed tea is not sufficient. In the research of black tea conducted by Hall *et al.* the MC of samples was limited in the range from 8.9% to 17.3% [9], and Sinija and Mishra detected the relationship between Fourier-Transform NIR spectroscopy and MC of green tea in the range of 3%–45% with 30 samples [10]. As the previous literatures only studied tea samples in a limited range of MC values, the relationship between MC of tea and spectral data should be more carefully studied. This research was conducted with fresh tea leaves, partially processed tea and manufactured tea with MC values in the range of 3.15%–71.40%.

Spectra from modern high throughput spectrometers often contain hundreds or thousands of spectral data points, and Vis/NIR spectra are characterized by generally overlapping vibrations of overtones and combination bands, in consequence these bands may appear to be non-specific and poorly resolved. So multivariate analysis plays a very important role in analysis of spectral data, such as principal component analysis (PCA), multiple linear regression (MLR), partial least squares regression (PLSR) and principal component regression (PCR). Especially, PCA, PLSR and PCR are all based on orthogonal transformation techniques, so these algorithms not only can greatly reduce the complexity of modeling, but also can eliminate the adverse effects caused by multicollinearity among spectral variables. However, PCA, PLSR, PCR and MLR can only deal with the linear relationship between spectral data and composition concentration, and the nonlinear information can hardly be calibrated by these linear models [13], when in fact, the absorbance often varies nonlinearly with concentration in multicomponent systems.

Nowadays, nonlinear algorithms including kernel principal component analysis (KPCA), artificial neural network (ANN) and least squares support vector machine (LSSVM) are frequently used for description of nonlinear phenomena [13–15]. Besides, wavelet transform (WT) shows great potential in the study of biological systems due to its merits in both space and frequency localization [16], exemplified in applications such as wind fields estimation [17], multi-spectral imaging classification [18], and soil spectral analysis [19,20]. Through decomposition of data in different scales and frequencies, the inherent structure and characteristic information may be discovered in wavelet decomposition coefficients [21,22]. Furthermore, it is easy to obtain the relationship between wavelet decomposition coefficients and original spectral data based on the clear decomposition structure of WT, which can be used to detect effective wavelengths for the composition, but few reports can be found in literature in relation to how to detect the effective wavelength for WT analysis.

The objectives of this study were: (1) to investigate the response of Vis/NIR diffuse reflectance spectroscopy toward MC of fresh tea leaves, manufactured green tea and partially processed green tea; (2) to perform and compare linear and nonlinear feature extraction algorithms for discovering the latent structure of spectral data, which included PCA, KPCA and WT; (3) to acquire characteristic wavelengths for determination of MC of tea based on WT.

## 2. Experimental Section

### 2.1. Materials

For sample diversity, three types of samples were collected, which included fresh tea leaves, manufactured green tea and partially processed green tea. The total number of samples was 738. The general information of samples was summarized in Table 1. Hereinto, the fresh leaves of type I were picked from five varieties of tea plants, and these samples were comprised of different tenderness leaves including young shoot, mature leaves and senescent leaves. The detailed information of samples in type I is shown in Table 2. Type II contained Xi-hu-long-jing tea of seven grades, and their detailed description is given in Table 3. Type III included eight kinds of partially processed green tea from eight processing procedures, as shown in Table 4.

In modeling, all 738 samples were divided into the calibration set and the prediction set with a ratio of 2:1. To avoid bias in subset partition, all samples were first arranged in an ascending order according to their respective MC values, then one sample was picked out from every three samples consecutively, resulting in 246 samples of prediction set, and the remaining 492 samples formed calibration set. The statistical information of *Y*-value of each set was shown in Table 5.

### 2.2. Spectra Acquisition and Reference Method for MC

**Figure 1.**
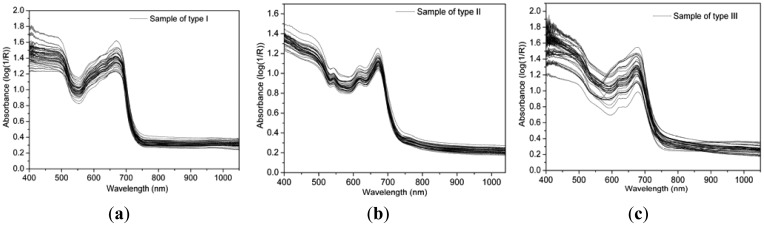
Vis/NIR diffuse reflectance spectroscopy of the samples.

In this study, a Vis/NIR spectroradiometer (FieldSpec®3, Analytical Spectral Devices, Inc., Boulder, CO, USA) was adopted for Vis/NIR spectroscopy acquisition. This spectroradiometer has high sensitivity in the range of 325–1,075 nm with a 512 photodiode array detector, while the field-of-view is 10°, the spectral resolution is 3.5 nm, and the interval of sampling is 1.5 nm. A 150 watt halogen lamp was used to provide uniform light in the visible and short-wave near infrared range. When scanning spectrum, the spectroradiometer was fixed on a tripod with 45° between the spectroradiometer axis and horizontal line, and fixed at approximately 100 mm above samples. After each sample was scanned, it was taken away to empty the position for the next sample, this movement might lead to a change in the measurement system. In order to reduce this influence, the spectroradiometer was calibrated every half hour by a 100-mm^2^ white standard panel with approximately 100% reflectance across the entire spectrum. So, relative reflectance was calculated with measurements from both the samples and the standard panel as shown in Figure 1. With respect to each sample, a mean spectrum was averaged by 30 scans. Besides, there were obvious noises at the beginning and the end of the spectrum, so only spectral bands of 400–1,050 nm were taken for further analysis.

The reference MC was measured by the gravimetric method according to the Chinese National Standard GB8304-87. In detail, every sample was heated in a constant temperature oven at 103 °C for 4 h, and weighed before and after the heating by an electronic balance with an accuracy of 0.0001 g. All the measurements were carried out in a room at approximate constant temperature of 25 °C and relative humidity of 40–55%.

### 2.3. Data Analysis

#### 2.3.1. Wavelet Transform

**Figure 2.**
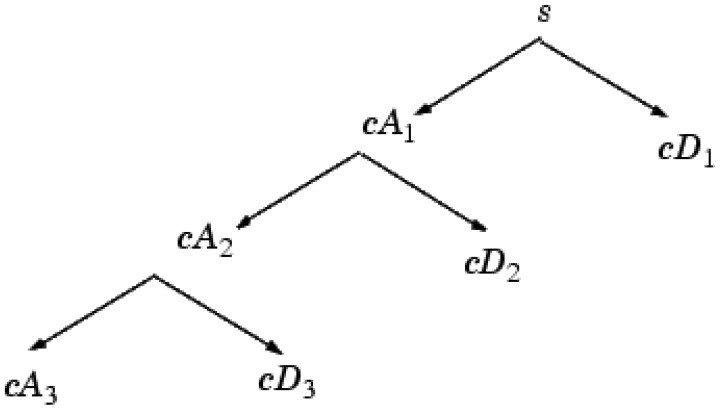
Structure of discrete wavelet decomposition at level 3.

WT enables the signal (spectrum) to be analyzed as a sum of functions (wavelets) with different spatial and frequency properties. The discrete WT (DWT) has the most popular application. The generated waveforms are analyzed with wavelet multi-resolution analysis to extract sub-band information from the non-stationary signals. The signal can be constructed accurately with the wavelet analysis using relatively small numbers of components [23,24]. The discrete WT decomposition structure was shown in Figure 2.

#### 2.3.2. Kernel Principal Component Analysis

KPCA is an extension of linear PCA using the kernel method technique, as shown by Schölkopf *et al.* [25]. Using a kernel, the originally linear operations of PCA are done in a reproducing kernel Hilbert space with a non-linear mapping. The idea of KPCA is to firstly map the original data *X* = [*x*_1_,…,*x_n_*], *n* = 1,…,*N*, into a high-dimensional feature space *F* using a nonlinear mapping φ: *R^P^*→*F*, and then the linear PCA is executed in *F* based on the mapped data φ(*x_n_*). In this study, the powerful kernel function of gaussian radial basis (RBF) is adopted for KPCA [25].

#### 2.3.3. Least Squares Support Vector Machine

Least squares support vector machine (LSSVM) is a least squares version of support vector machine (SVM) proposed by Suykens and Vandewalle [26]. In this version, the solution of a convex quadratic programming (QP) problem of the classical SVM is replaced with a set of linear equations of LSSVM, which greatly simplifies the computational complexity. LSSVM is a machine learning method based on statistical learning theory, which also possesses unique capability of SVM in solving problem with small observation, non-linear, and high-dimensional data.

#### 2.3.4. Implementation Steps

Before calibration, spectral reflectance was transformed in absorbance [log(1/R)] to establish the linear correlation between spectral data and concentration of composition. Then, spectral data were processed by three types of feature extraction algorithms including WT, PCA and KPCA, and then the synthetic variables from each algorithm were used as predictors. In this study, WT was implemented with wavelet function of Daubechies 5 (db5) at level 3. For KPCA, a RBF kernel was adopted for establishment of nonlinear mapping, the optimal sig2 (*σ^2^*) of 9,878 was obtained corresponding to the lowest mean squared error through a traversal optimization. Three regression models were respectively developed by PLSR, MLR and LSSVM. Hereinto, WT was implemented based on MATLAB 7.0 (The Math Works, Natick, MA, USA). KPCA and LSSVM were realized by MATLAB 7.0 coupled with the free LS-SVM v1.5 toolbox (Suykens, Leuven, Belgium). The Unscramble^®^ 9.7 package (CAMO PROCESS, AS, Oslo, Norway) was adopted for realization of PCA, PLSR and MLR.

### 2.4. Evaluation Index of Regression Model

The quality of the regression model was quantified by root mean squared error of calibration (RMSEC), root mean squared error of prediction (RMSEP), and the correlation coefficient (*r*) between the predicted and measured parameters [27]. A good model should have a low RMSEC, a low RMSEP, a high *r*, and a small difference between RMSEC and RMSEP [14].

## 3. Results and Discussion

### 3.1. Spectral Attributes of Tea Samples

Vis/NIR diffuse reflectance spectra of the three types of samples are shown in Figure 1. Similar contours were seen for all three types of samples. An obvious absorption peak was detected at 680 nm which was caused by the intense absorptivity of chlorophyll in the red light range. After 680 nm, the absorbance sharply declined as the wavelength increased from 680 nm to 750 nm. Then the absorbance was flat and low throughout the whole near infrared region. It could be found that the tea samples mainly absorbed the visible light in the range of 400–680 nm, especially at 680 nm. This phenomenon may be caused by the strong absorption of pigments in tea samples, while the absorptions of near infrared light (750–1,050 nm) were relative lower.

Except of the above similarities, many differences also existed in the spectra among the three types of samples. Comparing Figure 1(a) with Figure 1(b), there were many different absorptive responses within the range from 540 nm to 640 nm. In detail, two small absorption peaks were detected at 540 nm and 610 nm in Figure 1(b), but these absorptive responses did not exist in Figure 1(a). This phenomenon might be caused by the color change along with the variation of MC between type I and type II. The MCs of samples in type I were all bigger than 50%, while those in type II didn't exceed 7%, as shown in Tables 2 and 3. The big variation of MC caused by heating and drying led to huge concentration changes of chromogenic compositions in tea leaves. Former researchers have found that the chlorophyll *a* and chlorophyll *b* gradually degrade, and the contents of pheophytin *a* and pheophytin *b* increase in manufacturing process [28]. In type III, samples came from eight kinds of processing procedures, and the MCs were distributed in a broad range from 3.7% to 67% as shown in Table 4, so those curves were dispersing in Figure 1(c).

### 3.2. Extracting Characteristic of Spectral Data

**Figure 3.**
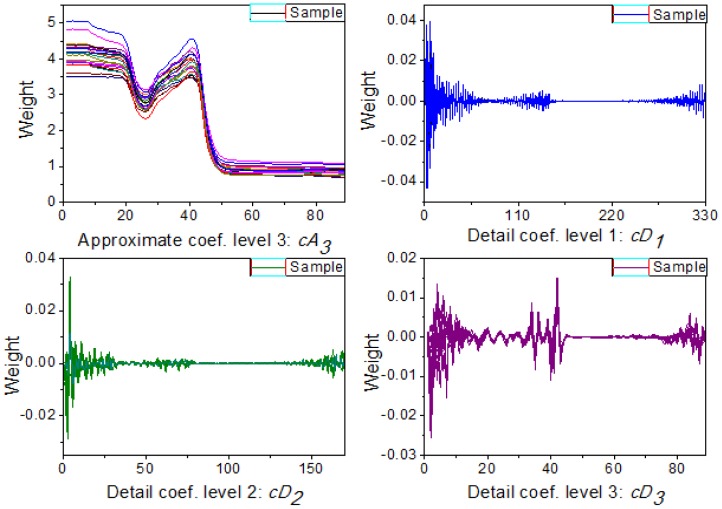
Wavelet decomposition coefficients by db5 at level 3.

**Figure 4.**
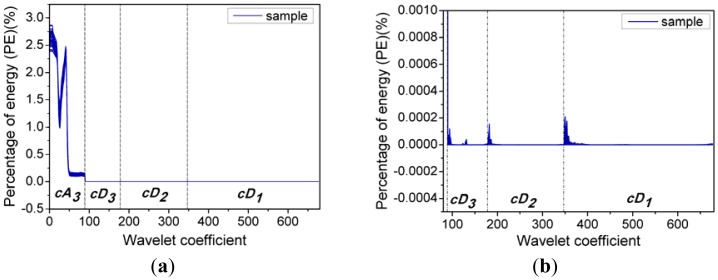
Energy distribution of wavelet coefficients.

Multi-signal wavelet decomposition was realized to expose the internal structure of all the spectral data of the 738 samples. After WT, the spectrum of each sample was decomposed to four sets of wavelet coefficients, including approximation coefficients *cA_3_* and detail coefficients *cD_1_*, *cD_2_*, *cD_3_* as shown in Figure 3. It could be found that *cA_3_* had the same trend with the original spectra, and it was very similar to the original spectra. While *cD_1_*, *cD_2_*, *cD_3_* contained much high-frequency information, especially in the beginning. In order to evaluate the information contained in the four sets of wavelet coefficients in this decomposition, the percentages of energy of the four sets of wavelet coefficients were calculated. And the energy percentages of wavelet coefficients for all the 738 samples were plotted in Figure 4.

Figure 4 shows the energy distribution of the wavelet coefficients including *cA_3_*, *cD_1_*, *cD_2_*, and *cD_3_*, where it can be seen that the energy percentages of the *cD_1_*, *cD_2_* and *cD_3_* are very close to zero, while the wavelet coefficients of *cA_3_* correspond to most of spectral energy. Furthermore, Figure 4(b) shows the energy distribution of *cD_1_*, *cD_2_*, and *cD_3_* in detail. It can be seen that their percentages of energy are very small, and there are relatively high-energy coefficients at the beginning of the three sets of detail coefficients. In other words, at the beginning of these detail coefficients contain a wealth of high-frequency information, which indicates that there is some high-frequency information at the beginning of the spectra [29]. Actually, due to potential system imperfection and limitation of spectroradiometer measurement, the scattering ray usually results in noise and disturbance at the beginning and the end of the spectral data [14], so this information at the beginning of these detail coefficients is likely caused by imperfections of the system and the spectroradiometer used in this research, so only approximate coefficients *cA_3_* are taken as characteristic features for further analysis.

**Figure 5.**
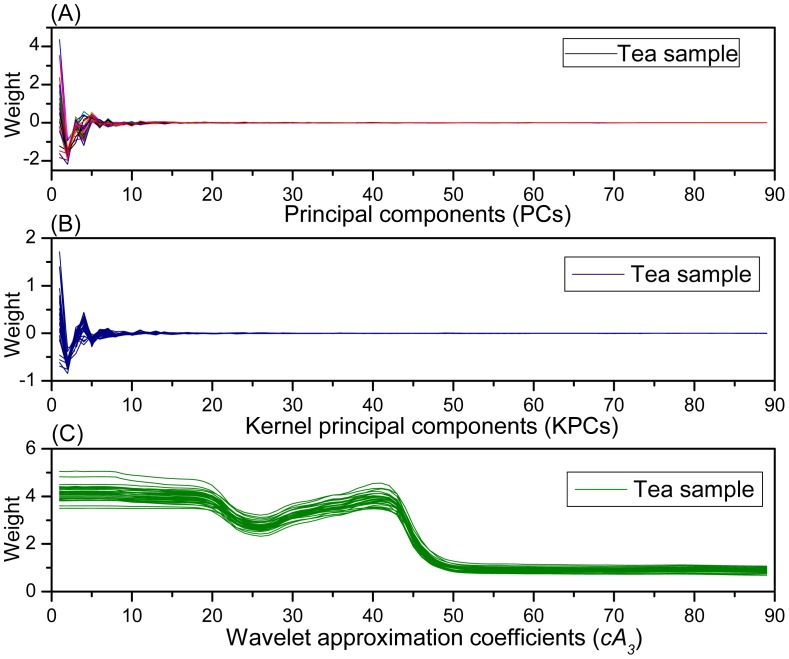
Description of tea samples in these new synthetic variable spaces, (**A**) in PCs space, (**B**) in KPCs space, and (**C**) in wavelet approximation coefficients (*cA_3_*) space.

Through feature extraction, WT, PCA and KPCA produced 89-dimensional new synthetic variables from original 651-dimensional spectral data respectively. Thus, samples can be represented with these new variables. Figure 5 shows the descriptions of tea samples in these new synthetic variable spaces. It can be found that the samples are described in the similar way by PCA and KPCA. Obviously, there are sharp peaks and valleys at the beginning of these curves in Figure 5(A,B), and then the curves gradually tend to 0, it can be concluded that most of the variance is centralized in the first tens of PCs and KPCs respectively. While in Figure 5(C) the 89-wavelet coefficients description of samples is very similar to the original spectral, indicating that the WT effectively captures the trend and characteristic information of the original spectra in low dimension.

### 3.3. Comparison of the Three Feature Extraction Algorithms

To evaluate the performances of WT, PCA and KPCA, three regression models (Models 1, 2 and 3) were respectively developed with the three sets of newly synthesized variables as predictors. Moreover, the original 651-dimensional spectra were also taken as predictor to develop regression model (Model 4). PLSR was adopted to establish regression models based on the full cross-validation method. The results of the above four models are shown in Table 6.

In Table 6, all four models afford excellent results. In detail, Model 4 outperforms Model 1 and Model 2 with much higher accuracy and smaller error. It can be concluded that there is much more useful information in the original spectral data than those in PCs and KPCs. In other words, PCA and KPCA result in loss of useful information through compressing the 651-dimensional spectral data into the 89-dimensional PCs and KPCs. Moreover, Model 2 is slightly better than model 1, which indicates that the nonlinear algorithm of KPCA catches more useful information than the linear algorithm of PCA. Model 3 based on the 89-dimensional *cA_3_* obtains the optimal result in the four models, which suggests that WT algorithm is more superior than KPCA and PCA algorithms for extraction of useful information. Especially, Model 3 is much better than Model 4, which indicates that the approximate coefficients of *cA_3_* not only cover the characteristic information of spectra, but also avoid the interference of noise in the spectra, and WT is a powerful tool for extraction of characteristic information from spectral data.

### 3.4. Obtaining the Optimal Regression Model

**Figure 6.**
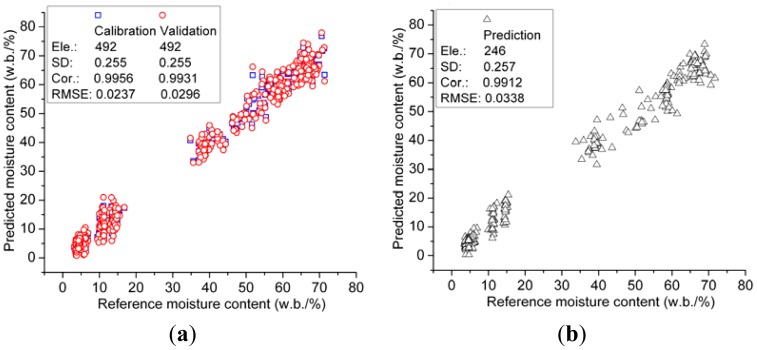
Scatter plot of reference *vs.* predicted of the optimal MLR Model 6 (**a**) calibration result and (**b**) prediction result.

As shown above, the 89-dimensional coefficients *cA_3_* were proved to be the optimal characteristics of spectroscopy, thus these coefficients were set as independent variables for further analysis. To obtain the optimal measurement, three regression algorithms including PLSR, MLR and LSSVM were adopted to develop regression models. Furthermore, LSSVM model was also based on RBF kernel function, and the kernel parameters of gam (*γ*) and sig2 (*σ*^2^) were optimized as 111,570 and 972.655 by grid-search which was a two-dimensional optimization procedure based on exhaustive search in a limited range [30]. The determination results of these three models are listed in Table 7. In detail, the MLR model obtains outstanding result with high correlation (*r*), and low root mean squared error (RMSE). Moreover, LSSVM model acquires excellent results in calibration stage, but the prediction results of the LSSVM model is slightly worse than that of the MLR model. And the PLSR model performs relative worse in both calibration and prediction stages comparing to MLR and LSSVM models. It may be concluded that the MLR model is the most proper description for the relationship between spectroscopy and MC. The results of the MLR model are plotted in Figure 6.

### 3.5. Detection of Fingerprint Wavelengths

**Figure 7.**
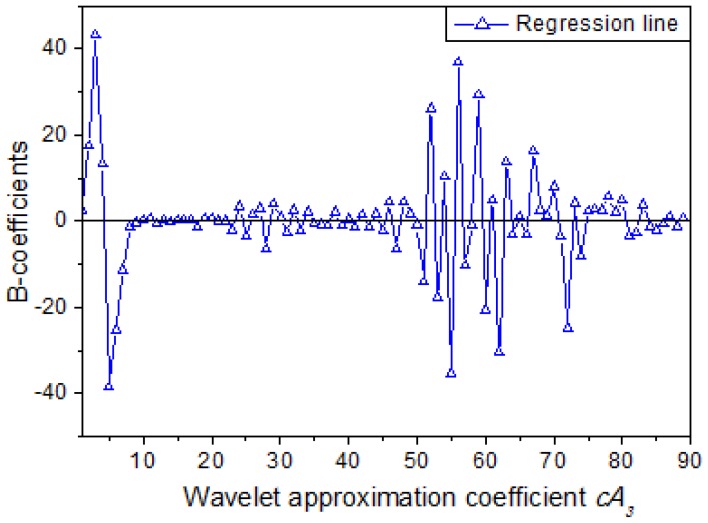
B-coefficients of the optimal determination Model 6.

In the MLR model, the relationship between wavelet coefficients *cA_3_* and response variable (MC) could be represented by a set of regression coefficients seen in Figure 7. It can be seen that the B-coefficients of many wavelet approximation coefficients are close to zero, and intense jagged peaks and valleys can be seen at the beginning and in the middle of the regression line. The B-coefficients represent the independent contributions of each independent variable to the prediction of the dependent variable. However, the amplitude of B-coefficients is related to the amplitude of the corresponding independent variables. So it is improper to detect fingerprint wavelength solely based on B-coefficients. In this manuscript, characteristic wavelength is obtained through combination of B-coefficients and experience as well as repeated attempting. Afterwards six determination models were established based on six sets of independent variables respectively, and the results are listed in Table 8.

**Figure 8.**
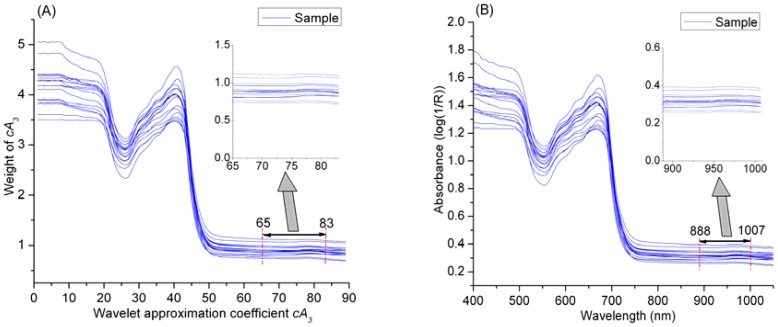
Reconstruction of approximation at level 3 (**A**) Wavelet approximation coefficients at level 3 and (**B**) Reconstructed signals.

In Table 8, Model 13 based on the 65th–83th coefficients of *cA_3_* obtains excellent determination results in both calibration and prediction stages, and the prediction accuracy (*r* = 0.991, RMSE = 0.034) is very close to that of Model 6 based on all the 89 coefficients of *cA_3_*. This phenomenon indicates that the *cA_3_* in the range of 65th–83th play an important role for determination of MC. What is the hidden meaning of this finding? As the wavelet approximation coefficients *cA_3_* is dimensionless, which is mathematic derived from original spectral data. Even though there is a clear linear formula relationship between wavelet coefficient and the MC of samples, the characteristic spectral absorbance of chemical bond O–H of water in the samples is obscure. However, there is a clear decomposition structure in WT, and WT has an outstanding reconstruction capability, so the relationship between spectral absorbance and MC might be detected by wavelet reconstruction. Figure 8 shows the reconstructed spectra, and the spectra in the range of 888–1,007 nm are generated from the *cA_3_* of 65th–83th based on wavelet reconstruction, so the absorption spectra of 888–1,007 nm might be the fingerprint wavelengths for characterization of MC. To test this hypothesis, a determination model based on these wavelengths (888–1,007 nm) was developed, and the correlation coefficient (*r*), RMSE of prediction and bias were 0.986, 0.046 and −1.450e^−02^ respectively. This result indicates that the spectra in the range of 888–1,007 nm are significantly correlated to MC of tea. This finding is corresponding to the strong and characteristic second overtone absorption position of O–H (960 nm).

## 4. Conclusions

The total results indicate that Vis/NIR diffuse reflectance spectroscopy data is significantly correlated to MC of tea, especially the wavelengths of 888–1,007 nm can be taken as fingerprint indicators of tea MC. This measurement method not only has high accuracy, but also can be applicable to a variety of tea leaves with different tenderness. Moreover, this model is suitable for several types of samples, including fresh tea leaves, manufactured green tea, and partially processed green tea in processing, which covers the range of MC values from 3.15% to 71.40%.

Linear transform algorithm and nonlinear transform algorithms (PCA, KPCA and WT) were all implemented to extract characteristic information from spectral data. Results indicated that the WT outperformed KPCA and PCA. It can be concluded that WT is a powerful tool for extraction of characteristic from spectral data. The capabilities of PLSR, MLR and LSSVM regression algorithms were investigated to establish determination models. The MLR regression model gave the optimal result. Moreover, the fingerprint wavelengths (888–1,007 nm) were detected by merged MLR with wavelet reconstruction. Overall results indicate that the Vis/NIR diffuse reflectance spectroscopy of tea is strongly affected by MC, it is feasible to measure MC of tea based on Vis/NIR diffuse reflectance spectroscopy with the conjunction of wavelet transform and multivariate analysis.

## Figures and Tables

**Table 1.** General information of the three types of samples.

**Types**	**Date**	**Number of Samples**	**Description**
I	2006.12.04	100	Fresh tea leaves
II	2007.09.12	70	Manufactured green tea
III	2008.10.12	568	Partially processed green tea

**Table 2.** Statistical information of moisture content (w.b., %) of samples in type I.

**Varieties**	**Range (%)**	**Mean (%)**	**SD** ^a^	**Number**
Longjing changye	54.662–68.421	62.906	0.038	20
Guangdong shuixian	66.029–69.792	67.715	0.011	20
Zisun cha	54.397–67.841	63.843	0.031	20
Maoxie	51.773–71.388	62.930	0.037	20
Longjing 43	56.410–68.889	63.958	0.040	20

a SD: standard deviation.

**Table 3.** Statistical information of moisture content (w.b., %) of samples in type II.

**Grades**	**Range (%)**	**Mean (%)**	**SD** ^a^	**Number**
Excellent grade	4.237–6.901	6.138	0.008	10
1 grade	5.075–6.644	5.558	0.005	10
2 grade	5.014–5.991	5.455	0.003	10
3 grade	5.312–6.050	5.737	0.002	10
4 grade	5.277–6.429	6.003	0.003	10
5 grade	5.521–6.286	5.896	0.003	10
6 grade	4.237–6.901	6.138	0.008	10

a SD: standard deviation.

**Table 4.** Statistical information of moisture content (w.b., %) of samples in type III.

**Procedure**	**Range (%)**	**Mean (%)**	**SD** ^a^	**Number**
Fresh leaves	61.347–71.723	67.021	0.023	74
Fixation	53.412–61.854	58.723	0.009	74
Rolling and cutting	39.567–60.506	51.327	0.049	72
Drying 1	33.780–44.404	38.766	0.018	74
Drying 2	12.082–16.838	14.191	0.008	70
Drying 3	9.459–11.556	10.916	0.005	76
Manufactured tea	3.148–4.638	3.728	0.002	58
Tea dust	4.171–5.214	4.613	0.002	70

a SD: standard deviation.

**Table 5.** Statistical information of moisture content (w.b., %) of samples in three data sets.

**Data sets**	**Range (%)**	**Mean (%)**	**SD** ^a^	**Number**
Calibration set	3.148–71.388	33.768	0.255	492
Prediction set	3.485–71.722	34.182	0.257	246
Total	3.148–71.388	33.906	0.256	738

a SD: standard deviation.

**Table 6.** Results of four PLS models corresponding to PCA, KPCA, WT and original spectral data.

**SN** ^a^	**FEA** ^b^	**IV** ^c^	**LV** ^d^	**Stages**	**Elements**	**Cor.** ^e^	**RMSE** ^f^	**Bias**
Model 1	PCA	89	10	Calibration	492	0.972	0.060	−1.802e^−09^
				Validation	492	0.969	0.063	−8.050e^−05^
				Prediction	246	0.961	0.072	−1.14e^−02^
Model 2	KPCA	89	11	Calibration	492	0.979	0.051	−4.649e^−09^
				Validation	492	0.976	0.046	−9.659e^−05^
				Prediction	246	0.966	0.060	−1.200e^−02^
Model 3	WT	89	13	Calibration	492	0.988	0.040	−2.770e^−07^
				Validation	492	0.985	0.044	1.634e^−05^
				Prediction	246	0.986	0.044	−4.800e^−03^
Model 4	non	651	13	Calibration	492	0.987	0.041	−1.637e^−08^
				Validation	492	0.985	0.044	−2.030e^−07^
				Prediction	246	0.980	0.052	−8.600e^−03^

a SN: Sequence number.

b FEA: Feature extraction algorithm.

c IV: Number of input variables.

d LV: Number of latent variables.

e Cor.: Correlation.

f RMSE: Root mean squared error.

**Table 7.** Results of three models corresponding to the three types of regression algorithms based on the wavelet approximation coefficients as predictors.

**SN** ^a^	**Alg.** ^b^	**Input**	**Stage**	**Elements**	**Cor.** ^c^	**RMSE** ^d^	**Bias**
Model 5	PLS	89	Calibration	492	0.987	0.041	−1.637e^−08^
			Prediction	246	0.980	0.052	−8.600e^−03^
Model 6	MLR	89	Calibration	492	0.996	0.024	−1.462e^−05^
			Prediction	246	0.991	0.034	−6.800e^−03^
Model 7	LSSVM	89	Calibration	492	0.999	0.013	−4.514e^−05^
			Prediction	246	0.986	0.044	−6.730e^−03^

a SN: sequence number.

b Alg.: regression algorithm.

c Cor.: correlation coefficient.

d RMSE: root mean squared error.

**Table 8.** Results of MLR regression models with different sets of wavelet approximate coefficients as independent variables.

**SN** ^a^	**Input**	**Stage**	**Element**	**Cor.** ^b^	**RMSE** ^c^	**Bias**
Model 8	2-7,51-57,59-60, 62-63,67,72	Calibration	492	0.951	0.079	−2.326e^−05^
		Prediction	246	0.909	0.107	−7.500e^−03^
Model 9	2-7,46-74	Calibration	492	0.982	0.048	−7.546e^−06^
		Prediction	246	0.978	0.054	−2.73e^−03^
Model 10	2-6,58-74	Calibration	492	0.969	0.063	−2.160e^−06^
		Prediction	246	0.965	0.067	1.220e^−04^
Model 11	58-74	Calibration	492	0.966	0.065	3.633e^−06^
		Prediction	246	0.968	0.065	−8.680e^−04^
Model 12	69-89	Calibration	492	0.986	0.043	−8.997e^−08^
		Prediction	246	0.983	0.051	−1.290e^−02^
Model 13	65-83	Calibration	492	0.992	0.032	1.103e^−06^
		Prediction	246	0.991	0.034	6.282e^−06^

a SN: Sequence number.

b Cor.: Correlation coefficient.

c RMSE: Root mean squared error.
